# Travelling Santa Problem: Optimization of a Million-Households Tour Within One Hour

**DOI:** 10.3389/frobt.2021.652417

**Published:** 2021-04-12

**Authors:** Tilo Strutz

**Affiliations:** Institute of Communications, Leipzig University of Telecommunications, Leipzig, Germany

**Keywords:** traveling salesman problem, fast heuristic, initial tour, hierarchical subdivision, UAV, TSP

## Abstract

Finding the shortest tour visiting all given points at least ones belongs to the most famous optimization problems until today [travelling salesman problem (TSP)]. Optimal solutions exist for many problems up to several ten thousand points. The major difficulty in solving larger problems is the required computational complexity. This shifts the research from finding the optimum with no time limitation to approaches that find good but sub-optimal solutions in pre-defined limited time. This paper proposes a new approach for two-dimensional symmetric problems with more than a million coordinates that is able to create good initial tours within few minutes. It is based on a hierarchical clustering strategy and supports parallel processing. In addition, a method is proposed that can correct unfavorable paths with moderate computational complexity. The new approach is superior to state-of-the-art methods when applied to TSP instances with non-uniformly distributed coordinates.

## 1. Introduction

The Traveling Salesman Problem (TSP) is a well-studied subject which has attracted researchers for decades. The main idea is to construct a tour with minimum length on which the salesman visits *N* cities (points) and gets back to its starting point. This kind of route optimization is a general problem that needs to be solved in many disciplines, such as vehicle navigation, logistics for delivery services, for cars and trucks, and more recently for unmanned aerial vehicles (drones) such as in digital farming. Many approaches toward finding optimal solutions have been proposed (see, for instance, Applegate et al., 2007) and dedicated software is freely available for academic use (Applegate et al., 2003,^1^; Helsgaun, 2009)^2^. These solutions rely mainly on the ideas of Lin (1965) and Lin and Kernighan (1973).

The research is nowadays typically redirected to TSPs with certain restrictions. One major problem that remained in conjunction with the TSP is its computational complexity. Optimal solutions exist for many problems up to several 10,000 points. However, seeking for optimality, when the tour comprises a million or more points, still takes too much time for practical usage despite the advances in computer technology.

**Figure 1:**
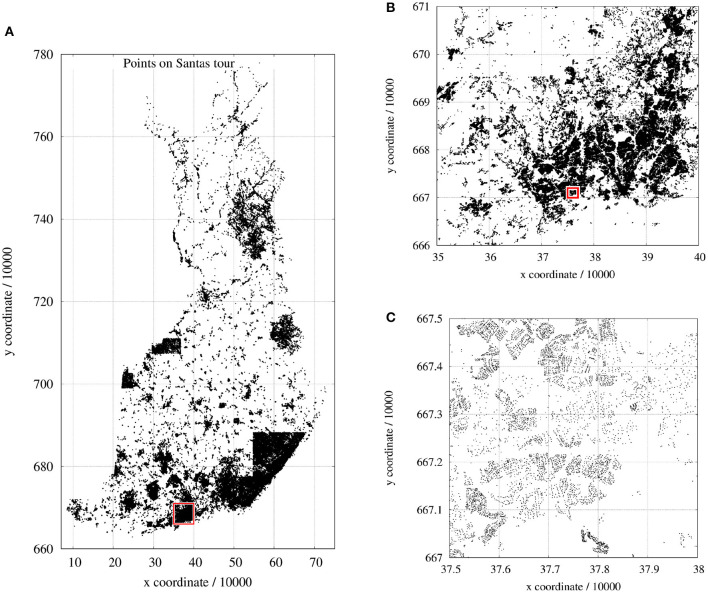
A special instance of TSP: the Traveling Santa Problem: **(A)** entire map of all 1.437.195 points; **(B)** close-up of **(A)**; **(C)** close-up of **(B)**.

The present paper had been triggered by a competition announced by the School of Computing, University of Eastern Finland^3^. Santa Claus has to deliver presents to the children in all Finnish families on Christmas Eve. He has to plan a tour passing 1,437,195 two-dimensional coordinates (Finnish National Coordinate System, see Figure 1) distributed all over Finland; and as a modern person, Santa may take advantage of unmanned aerial vehicles (UAVs) so that the entire tour also could be split into several tours. Two-dimensional Euclidean distances define the costs between pairs of points *p*_*i*_ and *p*_*j*_ and for the distances *d*(*p*_*i*_, *p*_*j*_) = *d*(*p*_*j*_, *p*_*i*_) holds.

Researchers had been invited to submit their source code that can be compiled and executed on a Linux computer. The target machine also had been specified with: Dell R920 with 4 x E7-4860 (total 48 cores), 1 TB, 4 TB SAS HD. This was a hint indicating the computational resources. However, Santa is usually somewhat tired after Christmas and he does not already start to plan his next tour at first of January. As soon as he feels rested again, the Christmas elves keep Santa Claus on his toes, and finally only 60 min remain for him to optimize his next tour on which he must visit all households.

The computational time of seeking optimal tours grows exponentially with increasing number of points. The optimization of a tour with more than one million points within 1 h requires heuristics which split the tasks in many sub-tasks with limited numbers of points for each optimization step. For this, we have to sacrifice the desire for an optimal tour.

One possible approach to achieve at least a good but sub-optimal tour is to subdivide all points in clusters, to process the clusters separately, and to combine the cluster tours (sub-tours) to an entire tour (Kobayashi, 1998). Also Muldera and Wunsch (2003) proposed a *k*-means clustering strategy with dynamically incremented number of clusters. Each cluster is separately optimized and these sub-tours are added in order of increasing distance from the origin, which is obviously only sub-optimal. This clustering idea describes a special formulation of the TSP that has been stated first by Chisman (1975) in application to a real-world warehousing problem. The points within individual clusters must be visited contiguously and the tour connects the clusters. That is, at first a coarse tour visiting all clusters has to be found followed by building sub-tours separately for each cluster. This idea was taken up by Helsgaun (2015) who presented a practical solution. However, in his set-up, the points already had to be arranged in clusters. Then the first point of each cluster is taken to build a coarse tour from cluster to cluster. An advanced version has been presented by Taillard and Helsgaun (2019). Based on a random selection of a certain number of points from the whole tour, a coarse tour is built. The remaining points are compared to the points of this coarse tour and are assigned to the closest ones followed by local optimization of each cluster. The initial tour generation of this approach has been publish separately by Taillard (2019) with focus on very fast processing.

Several researchers have addressed the question which tour quality can be achieved with a certain approximation algorithm. Christofides and Serdyukov have independently proposed an algorithm that guarantees solutions within a factor of 1.5 of the optimal tour length (excess ≤ 50%) if the instance is symmetric and obeys the triangle inequality (Christofides, 1976; van Bevern and Slugina, 2020). In Arora (1996), it was theoretically proven that a (1 + 1/*c*)-approximation can be achieved in *N*^*O*(*c*)^-time. This work has been extended by Mitchell (1999). While these considerations deal with theoretical bounds, our research aims at a practical trade-off between low complexity of the whole procedure (accounting for large instances) and low excess of the generated tour over the best known solution.

This paper proposes a new method for the generation of an initial tour that is as close as possible to the optimal tour within few minutes. This initial tour can be refined further by classical 2-opt or 3-opt operations. The proposed approach is not designed to yield optimal tours as no appropriate randomization, double-bridge kicks as introduced by Martin et al. (1992), or other suitable techniques are involved.

Since the target TSP instances have a 2D Euclidean metric given by coordinates, we can take advantage of this geometric information, as already pointed out by Bertagnon and Gavanelli (2020). For the initial tour generation we suggest a Double Local optimization With recursion (DoLoWire) procedure. Locality is considered in following two senses: (i) the approach is local regarding the geometric coordinates, which accelerates the clustering process, and (ii) it is local with respect to the sub-tour optimization of connected points. Here, the locality is represented by the sequence of adjacent points. The recursion of processing leads to clusters at different scales.

Compared to other software solutions, our underlying algorithm operates without linked lists and without tree structures making the software simple and transparent.

As the points to be visited are typically not arranged in well-defined clusters, cluster-based tour initialization causes disadvantageous paths. This paper additionally introduces a so-called “farmyard move,” which is able to tackle these problems by shifting a couple of points (the farmyard) from one to another of parallel paths.

Supporting a fast procedure, the proposal facilitates parallelizable steps of local optimization.

An earlier version of the new method achieved second place in the said Santa Claus competition. The winner was a LKH version^4^ submitted by Keld Helsgaun.

## 2. Methods

The discussion of the used methods starts with a well-known technique since it is the backbone tool at different steps of the proposed approach.

### 2.1. Backbone Optimizer

Given a circular tour with *N* = 10 points, we could number the points with (-0-1-2-3-4-5-6-7-8-9-), while point 9 is assumed to be connected also with point 0 (closed loop), and the order is defined using a vector **p** = (0 1 2 3 4 5 6 7 8 9). The vector **p** can be interpreted as a permutation vector determining the current order of points. If the tour length has not reached its minimum, the tour can be improved simply by permutations of the point order.

The most successful TSP solvers today are based on the Lin-Kernigham algorithm (Lin and Kernighan, 1973) and perform so-called *k*-opt operations, where *k* defines how many connections are removed to modify the order of points. For example, a 2-opt operation could cut the entire sequence between points 2 and 3 and between 6 and 7: **p** = (0 1 2 ∣ 3 4 5 6 ∣ 7 8 9). The only operation (or permutation) that can now be performed is a flip of the cut segment leading to **p** = (0 1 2 ∣ 6 5 4 3 ∣ 7 8 9) (Croes, 1958). Note that this is equivalent to flipping the outer segment: **p** = (9 8 7 ∣ 3 4 5 6 ∣ 2 1 0).

Permutations that are more complex could be achieved by combinations of flips, however, even if such combination of flips would lead to an improvement, one or more flips in this sequence of operations can lengthen the tour and the required permutation will not be completed without additional tricks. The algorithm gets stuck in a local minimum. This problem exists not only for *k* = 2 but also for *k*-opt operations with larger *k*.

The proposed method relies on 3-opt operations applying three cuts, which generate two adjacent segments as, for example, a first segment ∣ 2 3 4 ∣ and a second segment ∣ 5 6 ∣. These two segments can be modified by either reversing their order (flip) and/or by exchanging the segments position. Table 1 contains all possible variants of 3-opt operations.

**Table 1:** List of possible 3-opt operations based on a 10-points example.

**Operation #**	**Permutation**	**Remark**
0	0 1 ∣ 2 3 4 ∣ 5 6 ∣ 7 8 9	Original order
1	0 1 ∣ 4 3 2 ∣ 5 6 ∣ 7 8 9	Flip of first segment ∣ 2 3 4 ∣
2	0 1 ∣ 2 3 4 ∣ 6 5 ∣ 7 8 9	Flip of second segment ∣ 5 6 ∣
3	0 1 ∣ 4 3 2 ∣ 6 5 ∣ 7 8 9	Flip of both segments
4	0 1 ∣ 5 6 ∣ 2 3 4 ∣ 7 8 9	Exchange of both segments
5	0 1 ∣ 5 6 ∣ 4 3 2 ∣ 7 8 9	Exchange of both segments and flip of first
6	0 1 ∣ 6 5 ∣ 4 3 2 ∣ 7 8 9	Exchange of both segments and flip of second
7	0 1 ∣ 6 5 ∣ 4 3 2 ∣ 7 8 9	Exchange and flip of both segments

It is obvious that the operations 1 and 2 are only 2-opt permutations. This also applies to the last operation, as it is identical to a joint flip of both segments. Operation 3 combines two adjacent flips and could be helpful in some instances. Some authors have investigated which operations contribute most to the optimization success. The operations discussed in Sengupta et al. (2019), for example, are contained in the above table as special variants.

The modification of the tour depends on three parameters: the position *pos* of the first element of the first segment, the length *len*1 of the first segment, and the length *len*2 of the second segment. The optimal permutation (with respect to 3-opt) can be found by iteratively testing all possible modifications until a minimum tour length is reached, i.e., none of the possible modifications has led to any improvement in the last loop.

This brute-force 3-opt optimization is organized in a triple for-loop, see Listing 1. The outer for-loop defines the position *pos* of the first cut, i.e., it points to the first element of the first segment. The middle and the inner loop define the length of the first segment and the second segment, respectively. Both lengths are at least equal to one and their sum cannot go beyond the total number of points. Flipping the second segment is a forestalling of a first-segment flip at a later position. It is tested and performed only if no other operation shortens the tour. As soon as a tested operation would result to a shorter tour, this operation is performed. Afterwards, the length *len*2 of the second segment is increased if possible (inner loop) and so on. In total, there are (*N*^3^ − *N*)/6 different combinations of *pos*, *len*1, and *len*2 for a single scan making this technique only applicable to small *N*. The order of tested operations is with respect to Table 1: 7, 1, 5, 6, 3, 4, 2. It starts with the most complex operation.

**Listing 1:**
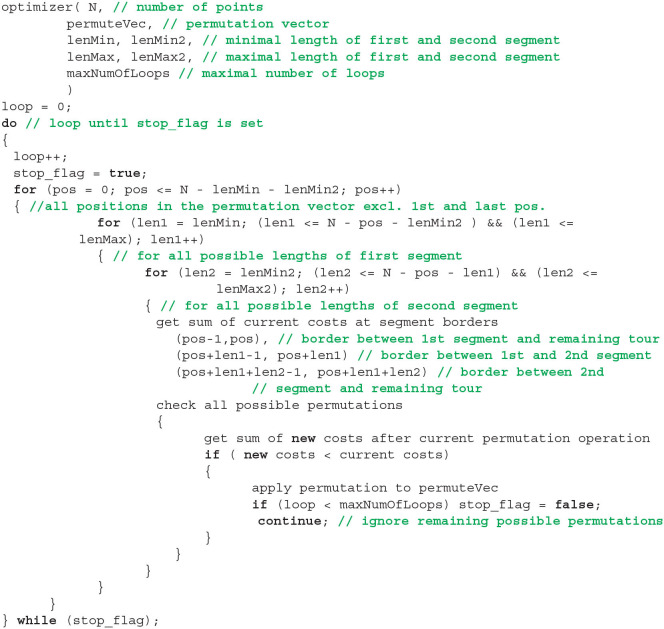
Pseudo code of 2/3-opt backbone optimizer. See text for details.

Testing an operation means that the distances between the points at cut positions must be compared before and after the permutation. As these distances must repeatedly be calculated, it would be favorable to pre-compute them one time in advance and to store them in a distance matrix. However, this requires *N*×*N*/2 elements to store and it is impossible to maintain such a matrix for large *N* as needed in the present traveling Santa problem.

If the optimizer runs in closed-loop mode, the first segment can start at the first position (*pos* == 0) of the permutation vector and the last element of the second segment may be identical to the last element (*pos* + *len*1 + *len*2 == *N*) of the permutation vector **p**. Since the proposed approach is based on the subdivision of the problem in sub-problems, which must be connected later on, the optimizer supports a second mode. It is a sub-tour mode, in which the first and the last element of **p** are assumed to be already positioned best, as they are connected to adjacent sub-tours. This mode can be seen as an open-loop mode with fixed start and end point. Hence, the optimization starts at *pos* = 1 and the second segment must not touch the last element: *pos* + *len*1 + *len*2 < *N*.

A variable *maxNumOfLoops* defines the maximum number of allowed iterations through the permutation vector. It limits the computational load to a reasonable amount depending on the tour that has to be optimized. The iteration stops when it either reaches this maximum number or no improving permutation has been found.

### 2.2. Cluster-Based Coarse Tour

The main idea of breaking the entire optimization problem in many smaller problems is based on clustering. We need a clustering method that covers all points and requires as few as possible processing steps for the assignment of points to clusters (or cells).

One of the state-of-the-art techniques is the *k*-means clustering. For a given number of cells, each is defined by its centroid, which is initialized with meaningful coordinates. The centroids are refined step by step by assigning each point to that cell to which it has the shortest distance. This assignment requires iterative comparison of all points with each cell and is far too time-consuming. To accelerate this assignment process, the following procedure is proposed.

**Figure 2:**
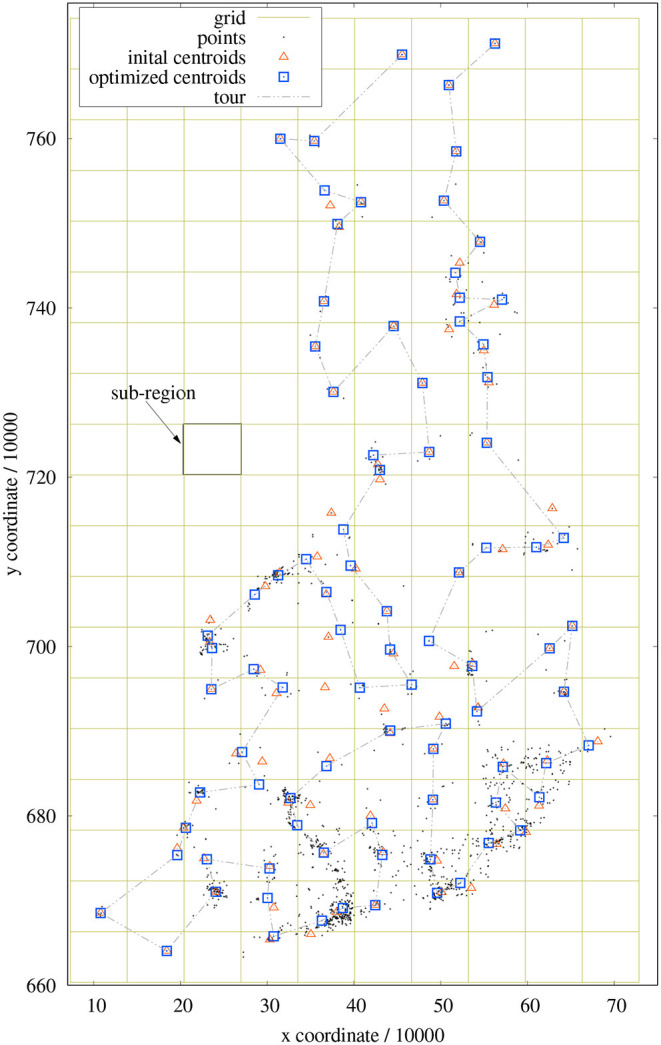
Example of sub-regions, cluster centroids, and coarse tour for a 1,437-points problem. Red triangles mark the centroids of the sub-regions; blue squares indicate the cluster centroids after new assignments.

The rectangular region enclosing all points with coordinates (*x*_*i*_, *y*_*i*_) is divided into a rectangular grid of *K*_*x*_ × *K*_*y*_ sub-regions, which are considered as clusters (see Figure 2). The grid size depends on the maximum number *maxCP* of allowed clusters (i.e., the maximum number of points in the coarse tour) as follows. At first, we have to identify the range of coordinates by

(1) $$rangeX=\underset{i}{\max}(x_{i})-\underset{i}{\min}(x_{i})\;rangeY=\underset{i}{\max}(y_{i})-\underset{i}{\min}(y_{i}).$$

In order to fulfill the two requirements

(2) $$\frac{K_{x}}{K_{y}}\approx \frac{rangeX}{rangeY}\text{ and }K_{x}\cdot K_{y}\approx maxCP,$$

we have to compute

(3) $$K_{x}=\left\lfloor q+0.5\right\rfloor$$

(4) $$K_{y}=\left\lfloor maxCP/q+0.5\right\rfloor$$

based on

(5) $$\begin{array}{r} q=\sqrt{maxCP\cdot \frac{rangeX}{rangeY}}. \end{array}$$

The centroid of each cluster is computed based on all points lying inside its sub-region. This assignment is achieved simply by the quantization of coordinates and without any comparison operations. Depending on the distribution of points, some sub-regions might not contain any point. Other sub-regions can contain points which are located at its borders and appear to be closer to the clusters of neighboring regions (see Figure 2). In order to improve the assignment of points to clusters, single points are moved from one cluster to an adjacent cluster (8-neighborhood) if it is closer to the centroid to this cluster. This procedure is somewhat similar to *k*-means clustering, however the number of comparisons is heavily reduced as only the centroids of neighboring clusters must be evaluated. This refinement is performed with a maximum of 32 iterations (empirically determined) as long as any re-assignment took place.

In this course, the cluster centroids adapt to the means of covered point coordinates. Some centroids even leave their sub-region and move into another region. It can also happen that, for example, the points of a cluster are distributed in such a way that all points are captured by neighboring clusters and finally no point remains in the current cluster. Such vanishing clusters merely shorten the length of the coarse tour to be optimized and do not harm the processing. This re-assignment procedure results in a better representation of the point distribution (in terms of initial tour length) (see also section 4). Unfortunately, this clustering method implies circular clusters and future investigations have to ascertain, whether other clustering techniques are even more appropriate.

The coordinates of the derived centroids are taken as points of a coarse tour to be optimized. The optimizer of section 2.1 is used with *maxNumOfLoops* = 5 and, as a result, we know afterwards in which order the clusters have to be visited. This information is important for the subsequent optimization of sub-tours.

Note that the value of *maxCP* is defined for the top-level (*level* = 0) of clusters. It should be chosen high enough to ensure a reasonable coarse tour but may not be too high as this would slow down the processing. If the algorithm goes into recursion, the coarse tours are allowed to be shorter without adversely affecting the tour quality. The optimal choice in terms of speed would be $maxCP_{\text{new}}\approx \sqrt{N}$ if *N* is the number of points per sub-tour. However, it turned out that more points per coarse tour benefit the final tour length. As a compromise, $maxCP_{\text{new}}=\min\left(maxCP,\left\lceil N^{0.7}\right\rceil \right)$ is chosen.

### 2.3. Sub-tour Optimization and Creation of Initial Tour

The points of each cluster form a segment (sub-tour) of the entire tour. Since it has to be ensured that adjacent sub-tours are connected in suitable manner, each list of sub-tour points is complemented with two end-points: one stemming from the preceding cluster and one from the succeeding cluster. The optimization is then performed using the sub-tour mode of the backbone optimizer described in section 2.1, that is, the optimizer performs open-loop operations and the two end-points are not allowed to move. The maximum number of loops is set to *maxNumOfLoops* = 5 because this leads to sufficient tour quality in most cases while keeping the computational cost low.

The approach of Taillard and Helsgaun (2019) applies a different method for sub-tours. It sticks to a closed-loop procedure and achieves the end-point fixation by setting the distance between the two end-points artificially to zero and by adding a penalty to the distances from an end-point to any other point. No points from adjacent clusters are considered.

In our approach, the selection of end-points is achieved by a pairwise nearest-neighbor search between all points of consecutive clusters. As only points from two neighboring clusters are involved, this technique rapidly leads to reasonable candidates and to minimal tour costs for the connections from one cluster to the next.

If the number of points of a sub-tour is larger than a predefined number of points *maxSP*, the division into sub-regions is recursively applied as described in the previous subsection. This can be interpreted as a hierarchical clustering procedure. Note that also the coarse tour generation now has to be treated as a sub-tour problem (open-loop and fixed end-points). The value of *maxSP* may be different from *maxCP*; the optimal choice of both thresholds depends on the speed requirements and the number of available cpu cores (see also section 3).

**Figure 3:**
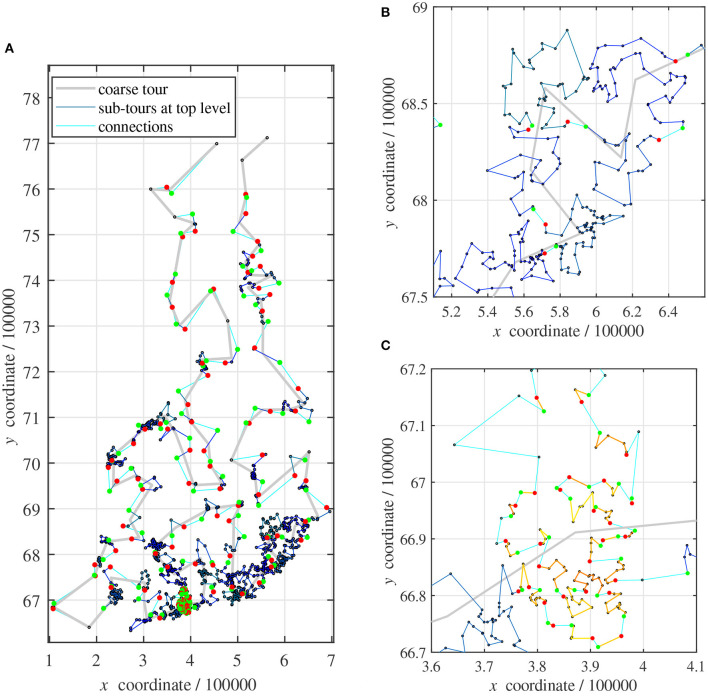
Sub-tours: **(A)** map of all 1,437 points; **(B)** close-up of **(A)**: sub-tours on top level; **(C)** close-up of **(A)**: sub-tours on second level.

As soon as all sub-tours have been optimized, on either top level or recursively, they can be merged to a full tour. In Figure 3A, each vertex of the coarse tour (pale thick line) corresponds to a cluster at top level.

The sub-tours of all clusters on top level are shown in Blue (thin dark lines). Their first sub-tour points are always marked with a red disk and the last points are indicated with a green disk, Figure 3B. The connections between clusters are drawn in Cyan (thin pale line).

This small example problem requires a single recursive clustering in a region with a very dense distribution of points. Figure 3C shows the corresponding sub-tours in yellowish colors. Sometimes, a cluster contains only one or two points. No sub-tours are plotted in these cases.

An analytical complexity estimation of the described method is difficult to accomplish because the points are not evenly distributed in the two-dimensional landscape and the resulting number and size of the clusters is unknown. Therefore, the complexity has to be determined empirically. The entire procedure of initial tour generation including time measures is investigated in section 4.1.

### 2.4. Refinement of Initial Tour

The second part of the proposed approach consists of several rounds of local optimizations. The entire tour whose creation has been discussed in section 2.3 is split in non-overlapping sub-tours (segments) of certain length *N* and each sub-tour is optimized separately. The algorithm takes care that the sub-tours contain disjunct sets of points.

If any of the sub-tours covering the whole tour could be improved, the processing starts again. In each loop, the start position of the first segment is randomly chosen, so that optimization across the sub-tour borders of the previous round is possible.

The optimization of each segment is again performed with the technique discussed in section 2.1 using sub-tour mode. The maximum number of loops within a single segment is set to *maxNumOfLoops* = 1. The idea is to access all segments as quickly as possible and hopefully achieve large improvements, rather than fully optimizing individual segments with only small successes at the same computational cost.

**Figure 4:**
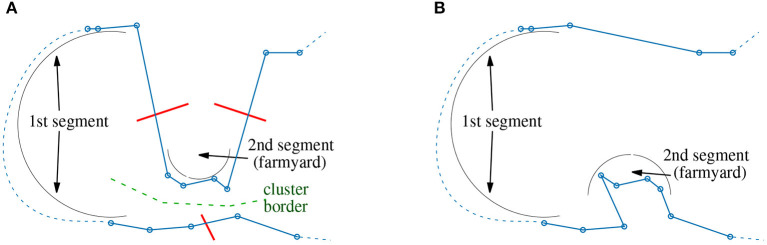
Farmyard move: **(A)** unfavorable assignment of points to clusters; **(B)** correction using a 3-opt operation with exchange of segments and flip of 2nd segment.

For the processing of segments, two different configurations are considered. The first setting is called “farmyard move” and is used for the correction of suboptimal cluster assignments. Sometimes the point assignment discussed in section 2.2 splits true clusters in an unfavorable manner (see Figure 4A).

Two parts of the tour are geometrically close together but along the sequence of points, they show a long distance (dashed line). The backbone optimizer has to use long segments otherwise the required 3-opt operation cannot be performed. However, long segments mean high computational costs. Fortunately, one of the segments typically consist of only few points (buildings) which are regarded as a farmyard. The tour can be improved by exchanging both segments and flipping the farmyard (Figure 4B). The computational efforts to find such improvement possibilities can be kept moderate using a combination of *lenMax*2 ≪ *lenMax* = *N*_1_, see Listing 1. So, a farmyard move is basically a 3-opt operation of type #6 (see Table 1), where the flipped segment is much shorter than the other segment to drastically reduce computational costs. The second configuration operates with the classical setting *lenMax*2 = *lenMax* = *N*_2_ and *N*_2_ ≪ *N*_1_. Both configurations are alternately applied. The farmyard-move configuration applies a reduced set of 3-opt operations. Only operations #6 and #1 from Table 1 are investigated saving some computations.

The default settings are as follows. At first, the farmyard move starts with segments of length *lenMax* = 10, 000 and farmyards with *lenMax*2 = 6 points. This round of optimization is followed by a regular configuration with segments of length *N*_2_ = 300. With each new round of farmyard moves, *lenMax* is increased by 10, 000 and the farmyard size by 4. The regular configuration continues with segments, which are lengthened in increments of 200. These increments possibly enable more improvements from round to round. This procedure is repeated until the maximum duration (1 h) is reached. Starting with relatively short segments, which are gradually lengthened, allows even slower computers to run several rounds of optimization within one hour.

## 3. Implementation Details

The software has been written in ANSI-C from scratch without using any special libraries or source code from other resources.

As the processing time is limited to one our, special focus is put on fast program execution. Ideally, the backbone optimizer described in section 2.1 can obtain the distances of points from a prepared distance matrix. However, it is not possible to provide a complete distance matrix as it requires *N*^2^/2 entries. A common strategy is to newly create the distance matrix for each separate sub-tour comprising only the points belonging to the current sub-tour (Taillard, 2019). A separate permutation vector also has to be allocated. The resulting permutation order must subsequently be transferred to the global permutation vector. This computational overhead is more than compensated by faster operations in the optimizer, when the distance matrix can be accessed instead of newly computing the distances for each single combination of *pos*, *len*1, and *len*2. The software limits the preparation of distance matrices to sub-tours up to 10,000 points. In respect to memory requirements, this is about the maximum possible when parallel optimization threads are taken into account.

This leads to the second possibility to speed up the computations. Since the optimization function typically processes non-overlapping sub-tours, several processes can be run in parallel in a multi-threading environment. The software determines the number of threads that can be run on all CPU cores and manages the calls of the optimization function accordingly. If the total number of threads is *T*, the main thread starts *T* − 1 parallel threads in maximum leaving some resources for the operating system.

The creation of the first coarse tour (at top level) runs in the main thread, i.e., the value of *maxCP* influences heavily the computational load at this part. As soon as the coarse tour and therewith also the sequence of the clusters is known, all corresponding sub-tours can be optimized in parallel. If a sub-tour is longer than *maxSP*, however, it cannot be optimized directly but must be processed in recursion. That means, at first a coarse tour through this cluster has to be generated. The required call of the backbone optimizer is done again in the main thread and is running in parallel to all previously started sub-tour optimizations. Since the current implementation does not embed the recursive execution in a separate thread, the calling function cannot start new threads for the optimization of subsequent sub-tours until the recursively called function is finished. From this point of view and with multi-threading in mind, it seems to be reasonable to set *maxSP* ≥ *maxCP* otherwise the available CPU cores are not efficiently utilized.

After all sub-tours have been optimized and merged to an initial tour, the complete tour is processed in non-overlapping segments. These segments can be optimized independently, so the backbone optimizer is called in multi-threaded mode again. In contrast to the initial tour generation, all segments now have the same length (either *N*_1_ or *N*_2_, depending on the configuration explained in the section 2.4). This leads to nearly identical run-times of the optimization processes and the CPU cores can be exploited in an efficient manner.

The cost function used for the calculation of point distances is implemented in a separate function and can be easily changed, if the distances are not Euclidean.

The memory consumption is linearly determined by the number of 2D coordinates which must be kept accessible during the entire runtime. This also applies to the permutation vector. The initial tour generation requires additional space for the quantized coordinates. All other necessary allocations depend on different program parameters and occupy only a fraction of the memory space taken up by the coordinates. The distance matrices are the only critical part. For safety reasons, the pre-calculation of the distance matrix is restricted to sub-tours with a maximum of 10^4^ points. Therefore, in typical settings, only the first round of farmyard moves benefits from this acceleration technique.

## 4. Investigations and Results

The following investigations have been undertaken on a Linux system with a state-of-the-art CPU (AMD Ryzen 9 3900x, 12 cores, 24 threads, 3.8 GHz clock rate). The source code has been compiled with gcc and the “-Ofast” option.

### 4.1. Optimization of the Initial Tour

**Figure 5:**
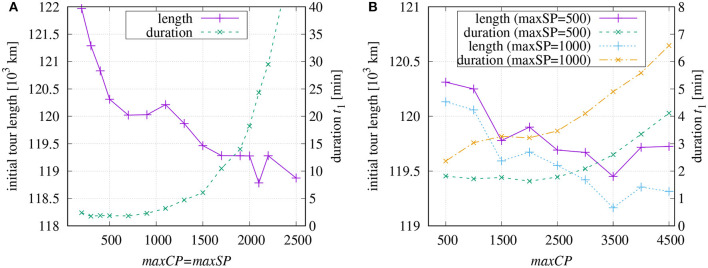
Computation times in minutes for building an initial tour and resulting tour length dependent on chosen values of *maxCP* and *maxSP*: **(A)** equal values *maxCP* = *maxSP*; **(B)** fixed value for *maxSP*.

As the software allows the parametrization of different aspects of the processing, it is of interest, which parameter combinations are most successful in terms of short tour length and low computational costs. Figure 5 shows the results of the initial tour generation depending on the chosen maximum number of coarse-tour points *maxCP* and the maximum number of points *maxSP* per sub-tour. As expected, the initial tour length^5^ decreases with increasing values *maxCP* and *maxSP*, Figure 5A, and the required computational time grows exponentially. It takes about 53 min to finish the initial tour when *maxCP* and *maxSP* are set equal to 2,500. The little peak around *maxCP* = 1, 100 and the dip at 2, 100 indicate that non-linear effects occur which might be caused by unfavorable routing of the coarse tour.

If *maxSP* is set to a fix value (Figure 5B), then the duration increases more slowly in dependence on *maxCP*. For longer sub-tours (*maxSP* = 1, 000), the tour lengths are somewhat shorter and, as expected, the processing lasts longer.

**Figure 6:**
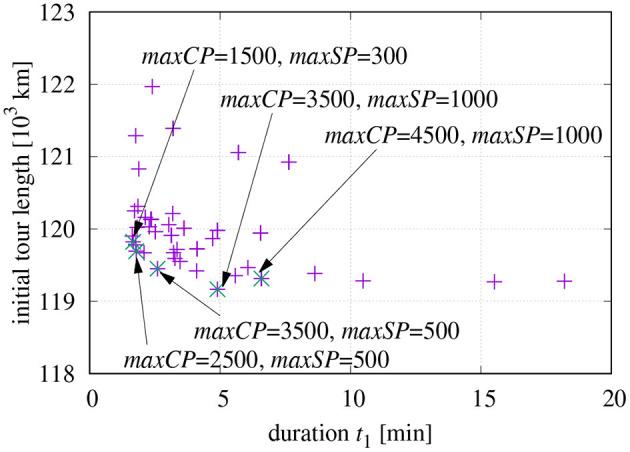
Initial tour length as a function of *t*_1_ and working points with good compromise between length and processing time.

It might be of interest, which combinations of *maxCP* and *maxSP* result in a good compromise of short initial tour and fast computation. The parameter space spanned by these two variables has been searched in the range 200… 2,500 and Figure 6 shows the resulting initial tour lengths as a function of *t*_1_. It seems that *maxCP*/*maxSP* = 1,500/300, is a good choice for *t*_1_ ≤ 3 [min] in the used set-up. The candidates 2, 500/500 and 3, 500/500 also seem to be promising; however, they turned out to be slightly inferior if the final optimization is included.

### 4.2. Final Optimization

The final tour optimization can use the remaining time *t*_2_ that has been left by the initial tour generation. So, we have to find an optimal share of the available *t*_1_ + *t*_2_ = 60 min. With regard to the diagrams in Figure 5, a reasonable tour can be found within *t*_1_ = 3 min. The faster the initial tour is generated, the more time is available to optimize the tour across the sub-tour borders. However, quickly generated but unfavorable paths of the initial tour can elude from being improved in the final optimization. A compromise is required.

**Figure 7:**
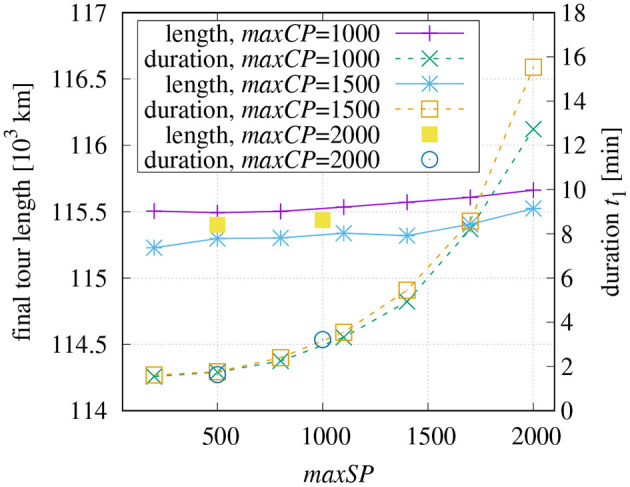
Final tour length after 60 min depending on parameters used for initial tour generation and fixed parameters for final optimization (*N*_1_ = 10,000 with *lenMax* = 6, *N*_2_ = 300).

Based on the investigation results shown in Figure 6, different combinations of parameters have been tested (see Figure 7). The final tour length is plotted for different *maxCP* values over varying values of *maxSP*. The default parameters for the final optimization have been chosen as explained at the end of section 2.4. The right vertical axis of Figure 6 additionally shows the time *t*_1_ required for the initial tour generation.

It can be seen that smaller maximum values *maxSP* for sub-tours benefit the global optimization in the chosen domain of definition. The reason probably lies in the shorter times of *t*_1_ which leave more time for the final optimization. The investigation also shows that a value of *maxCP* = 1, 500 is preferable over *maxCP* = 1, 000 as it leads to shorter tours despite the fact that the initial tour generation lasts slightly longer. It seems that *maxCP* = 1, 000 causes some unfavorable paths that cannot be removed later. The graph is complemented with two points using *maxCP* = 2, 000 showing that large values of this parameter not necessarily lead to better tours.

**Figure 8:**
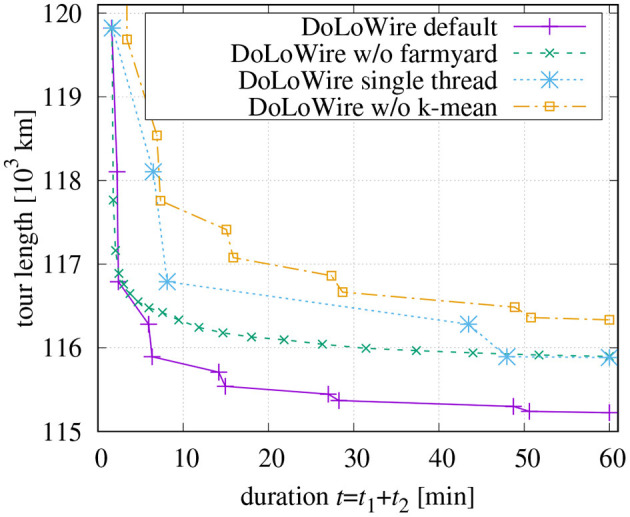
Influence of farmyard moves (initial tour with *maxCP* = 1, 500, *maxSP* = 300, *t*_1_ = 100 s), multi-threading, and cluster refinement.

The influence of the proposed farmyard move becomes apparent in Figure 8. The curves depict the tour lengths after successive steps of optimization. The first points are recorded after the initial tour generation. All other points belong to consecutive rounds of final optimization. There is a fast decay of the tour length after application of the first round because this is the first time where the optimization across the initial clusters takes place. This decay is more pronounced when the optimization start with segments of length of *N*_2_ = 300 (curve “w/o farmyard”) compared to farmyard moves with *lenMax*2 = 6 and *N*_1_ = *lenMax* = 10, 000 (curve “default”). However, without farmyard moves, the optimization converges very quickly, while using the farmyard moves in every second round of optimization pushes the tour toward shorter length. As the values of *N*_1_, *N*_2_, and *lenMax*2 get higher with each round of processing, the duration between the measured points also increases. The rounds of the farmyard moves take much longer, since they do not benefit from precomputed distances except for the first round with *N*1 = 10, 000.

Figure 8 also contains a curve showing the optimization behavior if only a single thread is used while all other parameters are kept. The markers (but the last) are at the same tour length values because identical processing is performed as in the multi-thread case. Following table compares the processing times:

**Table:** 

Processing	Times [s]		
step	Multiple threads	Single thread	Factor
Initial	98	101	1.03
Round 1	37	289	7.81
Round 2	9	97	10.78
Round 3	213	2,122	9.96
Round 4	24	269	11.21
Round 5	469	–	–

The measured times reveal that the initial tour generation profits only little by multi-threading. This has two reasons. First, the optimization of the coarse tour at top level has to be finished before the sub-tours can be processed. Secondly, the algorithm stops the processing on a certain level until a recursive call returns from a lower level. The optimization of subsequent sub-tours at the current level cannot be started in parallel. As *maxSP* < *maxCP* is chosen, the processing of previously started sub-tours is much faster than the generation of the next coarse tour. The second reason could be tackled by also encapsulating the recursive calls in a separate thread.

The table additionally shows the different behavior of the two configurations. The classic configuration (rounds with even number) utilizes only about 50% of the *T* − 1 = 23 CPU threads because there are only twelve physical cores. The program cannot benefit from hyperthreading since all threads require the same resources. The farmyard moves (rounds with odd number) benefit even less from multi-threading. One has to consider that not always all threads can be used. If, for example, there are *N* = 10^5^ points and the segment length is set to $N_{1}=10^{3}$, then *N*/*N*_1_ = 100 segments have to be processed. Given that 23 CPUs are available, then ⌊100/23⌋ = 4 times all CPUs are busy, while the remaining number of segments is only 100−4·23 = 8. Automatically, the average usage of CPUs is reduced.

The required duration for the farmyard-move configuration increases from round 1 to 3 much more than from 3 to 5. This is simply due to the already discussed fact that round 1 still can utilize a pre-calculated distance matrix without stumbling into memory shortage.

In section 2.2, a re-assignment of points based on a *k*-means like technique had been discussed. When this cluster improvement is skipped, not only is the length of the initial tour >122,000 km, but the computation time to generate the initial tour is also higher. This can be seen in Figure 8. Obviously, reasonable clusters ease the processing more than time can be saved than by not doing the re-assignment.

The said Santa competition requested four variants of optimization. In the first variant “closed-loop,” Santa returns home after visiting all points. In all other variants this is not necessary (“open-loop”). While in the second variant Santa may start from any location he wants, he must start from his home in Rovaniemi (first location in the dataset) in the third variant. In the fourth variant, Santa recruits some assistants (drone Santas) dividing the tour into *k* multiple parts that are solved by each drone separately.

The proposed method obtains following tour lengths:

**Table:** 

**1**	**2**	**3**	**4**	
**Closed loop **	**Open loop **	**Start in Rovaniemi **	***k*** = 2 ***Santas***	***k*** = 8 ***Santas***
115,224	115,188	115,224	115,153	114,989

As the proposed approach is only designed for the closed-loop mode, the other variants are simply created by searching the longest distance(s) between points of the final tour and cutting the tour at these points. The tour neighbors of Rovaniemi are closer than 1 km, so the (rounded) tour length is the same as for the closed-loop tour.

### 4.3. Comparison With State-of-the-Art Methods

Using large TSP instances from various sources, the performance of DoLoWire has been compared with two methods that can be parameterized such that they quickly output initial results and incrementally improve them.

The first method to compare with is based on an extended version of source code from Taillard (2019) (called POP+). The original POPMUSIC software was designed to run as fast as possible generating an initial tour. The default maximum length of sub-tours had been set to *t*^2^ = 225 points. With a budget of 60 min, POPMUSIC has been modified in two points: (i) parameter *t* has been changed to an empirical value of *t* = 70 points for our investigations and (ii) the implementation has been extended such that it iteratively optimizes the initial tour until the desired duration is reached, similar to the regular configuration described in section 2.4. The start length of segments to be optimized is set to *len* = 4, 000 and is increased by *len*/2 in each round.

The second selected method is LKH (Helsgaun, 2017). Using the default settings, LKH is not able to generate any tour for large instances within one hour, so special settings are required. The preprocessing step can be accelerated by using “INITIAL_PERIOD = 50.” There are different methods for generating an initial tour and “INITIAL_TOUR_ALGORITHM = GREEDY” yields the shortest tour for the Santa instance. In order to get a sufficient number of intermediate results, “MAX_SWAPS” is reduced to 2,000. “TIME_LIMIT = 3,600” forces the program to stop after one hour. However, this time limit does not include the preparation time. Additionally, “CANDIDATE_SET_TYPE = DELAUNAY” and “EXTRA_CANDIDATES = 4” have been suggested by Keld Helsgaun in application to the Santa instance.

The comparison should not only based on the Santa data set. However, there are few instances with 100,000 points or more that have already been used for testing optimization methods. Some have been published by DIMACS (Rutgers, New Jersey) in the course of a dedicated challenge. These data sets have been used among others by Taillard and Helsgaun (2019) and can be found online^6^. The University of Waterloo also maintains a dedicated web page with historical instances^7^. Especially the VLSI data set contains some large problems.

**Figure 9:**
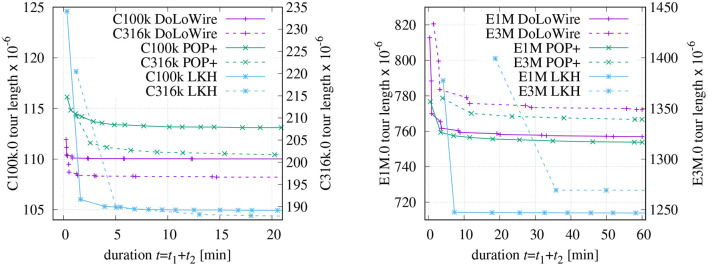
Progress of optimization for DIMACS instances (initial tour with *maxCP* = 1, 500, *maxSP* = 300). See text for details.

Figure 9 depicts the progress of optimization for different DIMACS instances. These data sets are artificially generated. While the points are uniformly distributed in the “E^*^.0” files, the “C^*^.0” data sets contain small clusters of points on a single level. As a result of the chosen parametrization in the proposed method, the duration of the farmyard-move configuration is always longer than the of the regular configuration. The sophisticated optimization heuristic of LKH produces superior results already after the first round of optimization. However, for instances with one million points and more, LKH requires distinctly more time than POP+ or DoLoWire to generate the initial and the first optimized tour as can be seen especially for E3M.0.

Table 2 compares the results with respect to the final tour length that have been obtained after 1 h of optimization. Along with the final tour lengths, the excess over the best-known solution is shown in percent. The first three rows contain the results for artificially generated instances with uniformly distributed point. The extended version of POPMUSIC shows here a slight advantage over DoLoWire. However, when applied to instances with clustered points, the proposed method performs distinctly better. The results for the VLSI instance “ara238025” are very close to each other. LKH achieves the best results for all instances with the settings discussed above. However, this is not the result of a clever initial tour generation method, but it is based on the sophisticated optimization techniques used.

**TABLE 2:** Results for different instances after 60 min of processing.

	**Size of**	**Length**				**% Excess**		
**Instance**	**instance**	**Best known**	**POP+**	**DoLoWire**	**LKH**	**POP+**	**DoLoWire**	**LKH**
E1M.0	100,000	713,187,688	753,807,836	757,343,554	713,914,467	**5.70**	6.19	0.10
E3M.0	3,162,278	1,267,318,198	1,339,204,602	1,348,907,974	1,269,251,701	**5.67**	6.44	0.15
E10M.0	10,000,000	2,253,088,000	2,389,639,423	2,431,057,748	^*^2,257,790,002	**6.06**	7.90	^*^0.21
C100k.0	100,000	104,617,752	113,054,381	110,103,932	104,874,011	8.06	**5.24**	0.24
C316k.0	316,228	186,870,839	201,135,304	196,653,823	187,395,021	7.63	**5.24**	0.28
ara238025.tsp	238,025	578,761	650,926	651,872	579,868	**12.47**	12.63	0.19
lra498378.tsp	498,378	2,168,039	2,383,112	2,357,782	2,171,629	9.92	**8.75**	0.17
lrb744710.tsp	744,710	1,611,232	1,816,607	1,806,674	1,614,552	12.75	**12.13**	0.21
Santa	1,437,195	108,416	119,054	115,224	109,161	9.81	**6.28**	0.69

Values of column “Best known length” are copied from Taillard and Helsgaun (2019) for DIMACS (^*^0.0) and from University of Waterloo (2020) for VLSI (^*^.tsp) instances. The best-known result for Santa has been generated by LKH. The tour lengths of POPMUSIC+ have been obtain by an extended version of source code from Taillard (2019) (^*^In order to get reasonable results for E10M.0 within 1 h, “INITIAL_PERIOD = 10” and “MAX_SWAPS = 100” had to be used).

### 4.4. Investigations on Complexity

**Figure 10:**
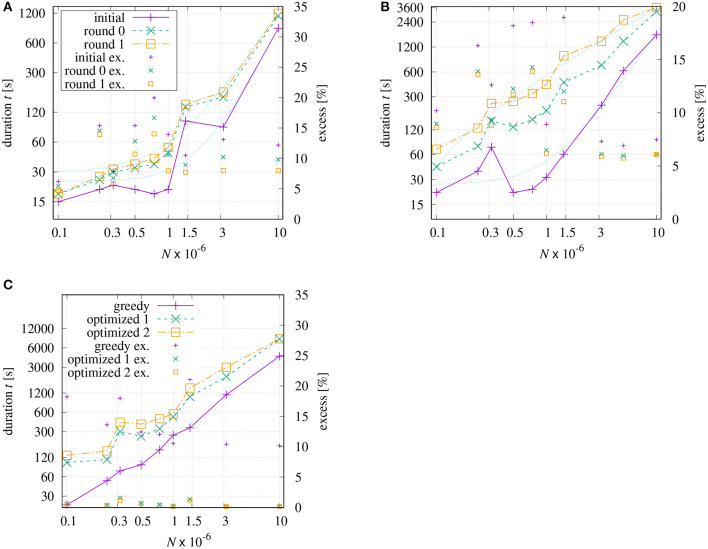
Processing time and excess (over best known solution) dependent on size *N* of all TSP instances from Table 2: **(A)** DoLoWire; **(B)** POP+ using same legend; **(C)** LKH.

With respect to initial tour generation, several researchers addressed the question which tour quality can be reached using a certain approximation algorithm. Christofides proposed an algorithm that guarantees solutions within a factor of 1.5 of the optimal tour length if the instance is symmetric and obey the triangle inequality (Christofides, 1976). In Arora (1996), it has been theoretically proven that a (1 + 1/*c*)-approximation can be achieved in *N*^*O*(*c*)^ time. For the proposed method DoLoWire it is difficult to derive such a bound theoretically. However, it is possible to obtain some indications based on experiments. Figure 10 shows the processing time and the excess (over best known solution) dependent on size *N* of all TSP instances from Table 2. In addition to the results after the initial tour generation, two curves are included showing the results after one and two rounds of final optimization, respectively. This seems to be reasonable as the pure initial methods do not optimize across the clusters, neither for DoLoWire nor for POP+.

The measured times for the initial tour generation of DoLoWire (Figure 10A) heavily depend of the presence on clusters and the required number of levels for clustering; that is why the Santa instance causes the peak at *N* ≈ 1.47 × 10^6^. When including the first rounds of final optimization, the curves *t* = *f*(*N*) get smoother. The thin dotted lines show fitted models $f\left(N\right)={\left(a_{1}\cdot N\right)}^{a_{2}}+a_{3}$. The model parameters have been derived using the fitting source code of Strutz (2016) to $a_{1}\approx 2.60\cdot 10^{-6}$, *a*_2_ ≈ 2.06, and *a*_3_ ≈ 24.1 for “initial” and $a_{1}\approx 8.52\cdot 10^{-6}$, *a*_2_ ≈ 1.59, and *a*_3_ ≈ 29.6 for “round 1,” where parameter *a*_2_ may give an indication of the likely underlying time complexity. The highest (and worst) excess equal to 20% can be observed in Figure 10A for instance “lrb744710.” After “round 1” the maximum excess is reduced to 14% (“ara238025” and “lrb744710”).

POP+ also shows some dependency on the structure of the processed instance (Figure 10B). The fitted model parameters are $a_{1}\approx 5.70\cdot 10^{-6}$, *a*_2_ ≈ 1.83,and *a*_3_ ≈ 26.1 for “initial” and $a_{1}\approx 3.61\cdot 10^{-3}$, *a*_2_ ≈ 0.78and *a*_3_ ≈ −57.9 for “round 1.” The latter results allows the assumption that POP+ has a linear complexity. The maximum excess is similar to DoloWire: 19.0% after initial tour generation (“Santa”) and 13.9% (“lrb744710”) after second round of final optimization. LKH generates the initial tour using the GREEDY algorithm which is distinctly more effective for instances with evenly distributed points, Figure 10C low excess values for E1M, E3M, and E10M. The first round of optimization drastically improves the tour. The subsequent optimization steps shorten the tour in relatively small steps. The worst excess using a greedy tour generation can be observed for the Santa instance with 21%.

## 5. Summary and Conclusions

A new method DoLoWire has been proposed that rapidly creates initial tours for symmetric instances of the traveling salesman problem. The approach uses grid-based clustering of coordinates in combination with a fast *k*-means like cluster refinement making the result almost independent of the initial point order. This method is combined with a segment-wise optimization of sub-tours. The initial tour generation is complemented with local refinements while the maximum duration is limited to 60 min. A special technique has been developed that is able to remove unfavorable paths of the initial tour over longer distances without exponential increase of the required processing time. Albeit originally developed for the Santa Claus challenge, DoLoWire has been proven also being successful when applied to standard TSP instances. In comparison to state-of-the-art methods, it generates initial tours faster and, in application to instances with unevenly distributed points, with shorter length. The used backbone optimizer lacks cleverness and this prevents better tours for the general case.

There are several ways to improve the current state of DoLoWire. One disadvantage is that the farmyard moves with large segments (*N* > 10,000) cannot benefit from the pre-calculation of distances. This threshold should not be fixed, but should depend on the available memory and the number of threads started in parallel.

With state-of-the-art hardware, DoLoWire generates initial tours (including two rounds of optimization across clusters) in <5 min for the investigated instances with *N* ≤ 3 · 10^6^. More time could be spent on sophisticated tricks either to improve the initial tour or to enhance the tour refinement than spending a mindless 55 min on 2/3-opt operations.

This leads to another option for improvement. The used brute-force 2/3-opt backbone optimizer can possibly be replaced with a more advanced optimizer that also can be run with less computational complexity. LKH currently sets the standard.

The proposed approach has been designed and tested for instances with two-dimensional Euclidean metric. Since the distance calculation is performed in a separate cost function, it is easy to adapt to other metrics if coordinates are given. Solving general TSPs is not possible with the proposed approach.

The implementation of the proposed method DoLoWire is accessible to support reproducible research (Strutz, 2021).

## Data Availability Statement

The original contributions presented in the study are included in the article, further inquiries can be directed to the corresponding author.

## Author Contributions

The author confirms being the sole contributor of this work and has approved it for publication.

## Conflict of Interest

The author declares that the research was conducted in the absence of any commercial or financial relationships that could be construed as a potential conflict of interest.

## Footnotes

^1^www.math.uwaterloo.ca/tsp/concorde/downloads/

^2^webhotel4.ruc.dk/keld/research/LKH/LKH-2.0.9.tgz

^3^cs.uef.fi/sipu/santa/

^4^http://akira.ruc.dk/~keld/research/LKH-3/

^5^All given lengths of the Santa instance are rounded to kilometers. The original coordinates are given in meters.

^6^http://mistic.heig-vd.ch/taillard/problemes.dir/tsp/tsp.html

^7^https://www.math.uwaterloo.ca/tsp/vlsi/index.html
